# Effects of mindfulness-based interventions on insight and stigma in patients with schizophrenia: a systematic review and meta-analysis

**DOI:** 10.3389/fpsyt.2025.1655057

**Published:** 2025-12-09

**Authors:** Xiaoxin Gao, Lixiu Wu, Guolin Jin, Kunqiang Yu, Xufang Yu

**Affiliations:** 1 Seventh Ward, The Second People’s Hospital of Lishui, Lishui, Zhejiang, China; 2 Department of Psychiatric Rehabilitation, The Second People’s Hospital of Lishui, Lishui, Zhejiang, China; 3 Central Laboratory, The Second People’s Hospital of Lishui, Lishui, Zhejiang, China

**Keywords:** mindfulness-based intervention, schizophrenia, insight, stigma, meta-analysis

## Abstract

**Objective:**

To systematically evaluate the impact of mindfulness-based interventions on insight and stigma in patients with schizophrenia.

**Methods:**

Randomized controlled trials (RCTs) and quasi-randomized controlled trials investigating the effects of mindfulness-based interventions on insight and stigma in schizophrenia patients were retrieved from databases including CNKI, Wanfang, VIP, CBM, PubMed, Cochrane Library, Embase, and Web of Science, with the search period spanning from inception to June 2025. Statistical analysis was performed using RevMan 5.2 software, with effect sizes expressed as standardized mean difference (SMD) and 95% confidence interval (CI). Heterogeneity was assessed using the I² statistic: a random-effects model was applied when I² ≥ 50% or P < 0.05; otherwise, a fixed-effects model was used. To evaluate the impact of study design on pooled effects, a case-deletion sensitivity analysis was conducted. Publication bias was assessed using funnel plots and Egger’s test. Intervention types included mindfulness-based stress reduction, mindfulness-based cognitive therapy, group mindfulness therapy, and mindfulness-informed psychoeducation/skills training, with intervention durations ranging from 4 to 24 weeks.

**Results:**

A total of 11 RCTs and 2 quasi-randomized controlled trials involving 2,899 patients were included. The analysis revealed that the intervention group exhibited significantly better insight (SMD=1.05, 95% CI=0.67–1.43, P < 0.00001) and lower stigma (SMD=-0.81, 95% CI=-1.00 to -0.63, P < 0.00001) compared to the control group.

**Conclusion:**

Mindfulness-based interventions can significantly improve insight and reduce stigma in patients with schizophrenia, holding important clinical implications. Specifically, they may enhance treatment adherence and rehabilitation participation, decrease self-stigma, and promote the restoration of social functioning. Higher-quality, long-term follow-up randomized controlled trials (RCTs) are still needed to further validate and refine the evidence.

**Systematic Review Registration:**

https://www.crd.york.ac.uk/PROSPERO/, PROSPERO identifier CRD420251172478.

## 1 Introduction

Schizophrenia is a complex chronic mental disorder characterized primarily by positive symptoms (e.g., hallucinations, delusions), negative symptoms (e.g., affective flattening, social withdrawal), and cognitive dysfunction (1). Furthermore, affective symptoms (such as depression, anxiety, and impaired emotion regulation) and psychomotor abnormalities (including psychomotor retardation/agitation, and even features associated with catatonic syndrome) are also prominent in a considerable proportion of cases and are closely linked to functional prognosis and relapse risk (2). In terms of functional outcomes, impaired insight and stigma are key factors influencing treatment adherence, social functioning, and rehabilitation in patients (3, 4). Insight refers to the patient’s awareness and acceptance of their illness, with poor insight being closely associated with treatment refusal, increased risk of relapse, and decline in social functioning (5). Meanwhile, stigma further exacerbates patients’ social avoidance, low self-esteem, and depressive mood, thereby forming the so-called “insight–depression/stigma paradox”: that is, higher levels of illness awareness may paradoxically be accompanied by greater self-stigma and emotional distress in some patients (6). Although antipsychotic medications can alleviate symptoms, traditional pharmacological treatments have limited efficacy in improving insight and reducing stigma, highlighting the urgent need to explore effective psychosocial interventions.

In recent years, Mindfulness-Based Interventions (MBIs) have demonstrated feasibility, safety, and potential benefits in the treatment of schizophrenia (7). Mindfulness training emphasizes non-judgmental awareness of present-moment experiences and may indirectly enhance patients’ illness awareness by improving emotion regulation, reducing cognitive fusion, and fostering self-acceptance. Cognitive fusion refers to the psychological process wherein individuals tend to treat transient thoughts or emotional experiences as objective facts or intrinsic aspects of the self, thereby becoming dominated by them. By reducing this tendency toward cognitive fusion, mindfulness training facilitates a more flexible relationship with internal experiences, thereby improving emotional regulation and illness perception (8). For instance, adapted versions of Mindfulness-Based Cognitive Therapy and Stress Reduction programs have been employed to alleviate emotional symptoms and enhance social functioning in individuals with schizophrenia (9). Additionally, preliminary evidence suggests that mindfulness training may reduce self-stigma by mitigating self-criticism and enhancing psychological flexibility (10).

However, existing research findings exhibit heterogeneity, with some studies reporting non-significant effects of mindfulness interventions on insight, and the impact on stigma has yet to be systematically evaluated. In previous research, a randomized and observational study provided preliminary support for the aforementioned mechanisms and clinical effects: group mindfulness-based interventions in inpatient populations showed positive signals in terms of feasibility and preliminary clinical outcomes (11); a cross-sectional study demonstrated systematic associations between mindfulness levels and process variables such as cognitive fusion and self-compassion (12). At the biological level, mindfulness and empathy may involve modulation of the oxytocin pathway (13), while psychological flexibility is also systematically correlated with symptom severity (14). Concurrently, recent systematic reviews and practice guidelines have offered a more comprehensive methodological reflection on the efficacy, indications, and safety boundaries of MBIs in populations with schizophrenia spectrum disorders (SSD) (15, 16). Beyond standalone psychological interventions, augmentation treatment approaches are emerging. For instance, an initial double-blind randomized controlled trial combining oxytocin with group mindfulness demonstrated potential synergistic effects on empathy and negative symptoms (17).

Therefore, this study aims to employ systematic review and meta-analysis, incorporating randomized controlled trials (RCTs) and quasi-experimental studies (quasi-RCTs), to clarify the interventional effects of MBIs on insight and stigma in patients with schizophrenia, thereby quantifying the evidence to define the impact of MBIs on insight and stigma and support personalized rehabilitation interventions for schizophrenia.

## 2 Methods

### 2.1 Inclusion and exclusion criteria

#### 2.1.1 Inclusion criteria

##### 2.1.1.1 Study type

Randomized controlled trials (RCTs); given the limited number of rigorous RCTs on psychosocial interventions in the schizophrenia population and the numerous ethical and feasibility constraints in inpatient/acute-phase samples, this study additionally incorporated quasi-randomized controlled trials (quasi-RCTs) to expand the evidence base and avoid evidence gaps. The influence of quasi-RCTs on conclusions was strictly controlled through stratification and sensitivity analyses, with no restrictions on blinding implementation. Language was limited to Chinese and English.

##### 2.1.1.2 Participants

Patients diagnosed with schizophrenia (18), regardless of age, gender, or ethnicity.

##### 2.1.1.3 Interventions

(1) The intervention group received mindfulness-based psychological/cognitive/stress reduction interventions: structured programs with mindfulness training as the core component, typically including Mindfulness-Based Stress Reduction (MBSR), Mindfulness-Based Cognitive Therapy (MBCT), Mindfulness-Based Group Therapy (MBGT), mindfulness-informed psychoeducation/skill training (e.g., MBPP/MPGP, MBPST), and hybrid (in-person + self-guided) MBCT.

(2) The control group received treatment as usual.

##### 2.1.1.4 Outcome measures

Insight; Stigma. Included studies had to report at least one of the above outcomes.

#### 2.1.2 Exclusion criteria

(1) Studies with insufficient data, duplicate publications, or unavailable full texts.

(2) Reviews, conference abstracts, case reports, or animal studies.

### 2.2 Search strategy

Computerized searches were conducted in the following databases: China National Knowledge Infrastructure (CNKI), Wanfang, VIP, China Biology Medicine (CBM), PubMed, Cochrane Library, Embase, and Web of Science. The search period spanned from inception to June 2025. The search terms used were: “Schizophrenia” OR “Psychotic disorders” OR “Psychosis”;”Mindfulness” OR “mindfulness-based stress reduction”. Using a combination of subject headings and free-text terms, we conducted a comprehensive search that included the references of the incorporated literature. The search strategy is illustrated using PubMed as an example: (((Schizophrenia[MeSH Terms]) OR (Psychotic disorders[Title/Abstract])) OR (Psychosis[Title/Abstract])) AND ((Mindfulness[MeSH Terms]) OR (Mindfulness-based stress reduction[Title/Abstract])) AND (Humans[Mesh]). The protocol for this systematic review and meta-analysis was registered on the International Prospective Register of Systematic Reviews (PROSPERO) (Registration number: CRD420251172478). The design, conduct, and reporting of this review adhered to the Preferred Reporting Items for Systematic Reviews and Meta-Analyses (PRISMA) guidelines.

### 2.3 Literature screening and data extraction

Literature screening initially involved automated deduplication using the NoteExpress reference management system, followed by manual deduplication and cross-verification by two researchers based on title, author, journal name, publication year, DOI, or registration number to enhance the accuracy of duplicate removal. After deduplication, preliminary screening was conducted by reviewing titles and abstracts. Articles that passed the initial screening were further evaluated by reading the full text, and those that did not meet the inclusion criteria were excluded.

Data extraction was performed independently by two researchers based on the predefined inclusion and exclusion criteria. In cases of disagreement between the two researchers, a third researcher was consulted for adjudication. The extracted data included the title, authors, publication date, study type, age of participants, sample size, intervention methods, and outcome measures.

### 2.4 Quality assessment of literature

Two researchers independently evaluated the quality of the included studies using the Cochrane Risk of Bias Tool (Handbook 5.1.0) (19) for randomized controlled trials (RCTs) and the Joanna Briggs Institute (JBI) Critical Appraisal Checklist for quasi-RCTs (2016) (20) from the JBI Evidence-Based Healthcare Center, Australia. Among them, the RCT evaluation indicators assess the following aspects: the specific methods and procedures for random sequence generation; the approach used to conceal allocation sequences; the blinding of all study subjects and interveners; the blinding of assessors; the completeness of outcome data and whether missing data were reported; the presence of selective reporting of either positive or negative outcomes; and other potential biases. The evaluation criteria for quasi-RCTs include nine items: causal relationship in the study, baseline comparability, whether other measures are identical across groups, whether a control group is established, diversity in outcome measurement before and after intervention, completeness of follow-up, consistency in outcome measurement, reliability of measurement methods, and appropriateness of data analysis methods. Studies that fully meet these criteria are rated as Grade A; those that partially meet them are rated as Grade B; and those that do not meet them at all are rated as Grade C. If the assessments by two researchers are inconsistent, a third researcher will be consulted to reach a final decision.

### 2.5 Statistical methods

The analysis was performed using RevMan 5.2 statistical software, with clinical heterogeneity assessed by the I² test. When P ≥ 0.05 and I² < 50%, it indicated no statistical heterogeneity among the studies, and a fixed-effects model was applied for the meta-analysis. Conversely, if statistical heterogeneity was present, potential sources of variation were first analyzed. If no significant clinical differences were identified and no definitive statistical source of heterogeneity could be determined, a random-effects model was used for the meta-analysis. Since the insight and stigma indicators included in this study were continuous variables, the standardized mean difference (SMD) and its 95% confidence interval (CI) were used as the effect measures. A P-value < 0.05 was considered statistically significant.

To evaluate the impact of study design on the pooled effect, we performed a case-deletion sensitivity analysis: after excluding the study from the pooled analysis, we re-estimated the effect size and heterogeneity (reporting SMD, 95% CI, I², and the p-value for Q). If heterogeneity fell below the threshold (I² < 50%), the pooled results after exclusion were presented using a fixed-effects model as per the protocol, serving as evidence of robustness.

## 3 Results

### 3.1 Basic information of included studies and quality assessment results

**Figure 1:**
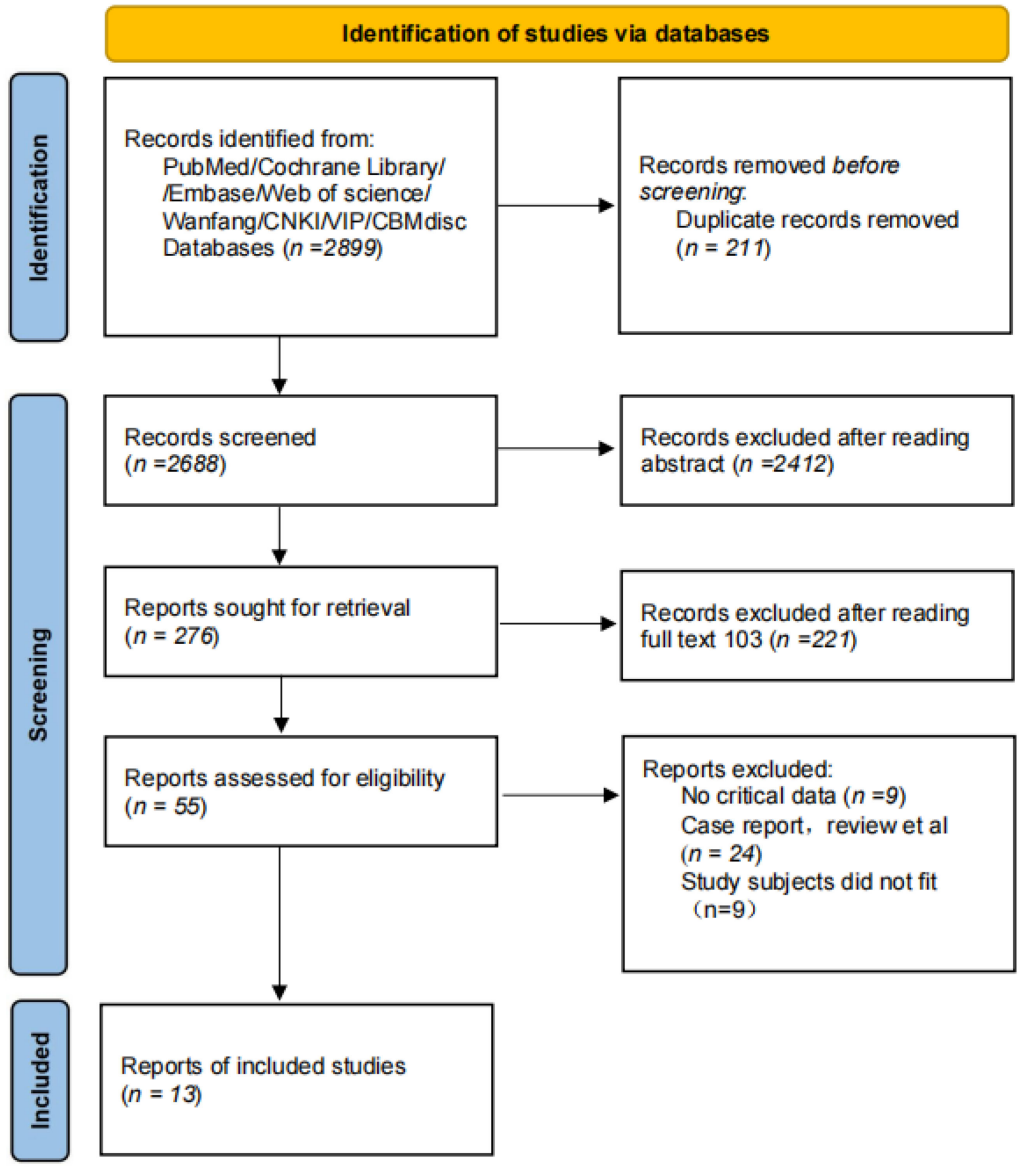
Literature screening flowchart.

**Figure 2:**
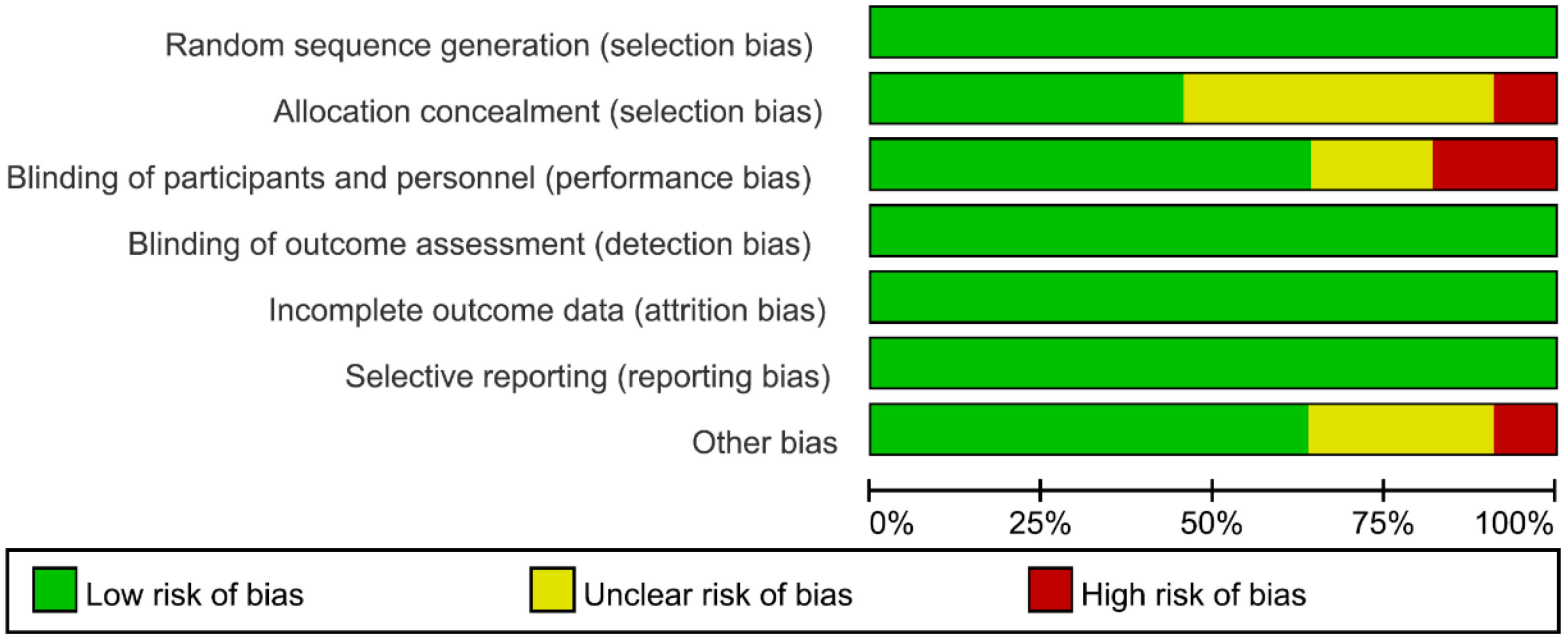
Risk of bias assessment graph for included RCT literature.

Through database searches, 2,899 articles were initially identified, from which 13 studies (21–33) (comprising 8 English and 5 Chinese publications) were ultimately included. These studies involved a total of 1,224 patients with schizophrenia (599 in the intervention group and 625 in the control group). The literature screening process is illustrated in **Figure 1**, and the basic characteristics of the included studies are summarized in **Table 1**. Quality assessment revealed that 6 studies were rated as Grade A and 7 as Grade B, with the results detailed in **Table 2** and **Figure 2**.

**Table 1:** Quality assessment results of included studies.

Items	Yılmaz2018 (23)	Yüksel2021 (24)
1.Was the causal relationship in the study clearly elucidated?	Yes	Yes
2.Were the baseline characteristics comparable between groups?	Yes	Yes
3.Apart from the intervention under investigation, were other measures received by each group identical?	Yes	Yes
4.Was a control group established?	Yes	Yes
5.Were outcome measures assessed multidimensionally both before and after the intervention?	Yes	Yes
6.Was follow-up complete? If incomplete, were dropouts reported and addressed with appropriate measures?	Yes	Yes
7.Were outcome measures for all study subjects assessed using identical methods across groups?	Yes	Yes
8.Were the measurement methods for outcome indicators reliable?	Yes	Yes
9.Were the data analysis methods appropriate?	Yes	Yes
	A	A

**Table 2:** Basic characteristics of included literature.

Author & year	Country	Study type	Sample size		Age		Intervention method		Intervention duration	Outcome measures
			Control group	Intervention group	Control group	Intervention group	Intervention group	Control group		
Dai2024 (21)	China	RCT	76	78	45.97 ± 8.67	45.67 ± 9.03	Mindfulness-Based Cognitive Intervention	Routine Care	8weeks	②
Çetin2018 (22)	Turkey	RCT	80	55	–	–	Mindfulness-Based Psychological Intervention	Routine Care	4weeks	①
Yılmaz2018 (23)	Turkey	quasi-RCT	24	21	34.58 ± 7.50	41.71 ± 14.65	Mindfulness-Based Psychological Intervention	Routine Care	8weeks	①
Yüksel2021 (24)	Turkey	quasi-RCT	19	19	–	–	Mindfulness-Based Psychological Intervention	Routine Care	11weeks	①
Chien2014 (25)	China	RCT	35	36	26.00 ± 8.50	25.10 ± 6.80	Mindfulness-Based Psychological Intervention	Routine Care	24weeks	①
Chien2019 (26)	China	RCT	56	56	25.40 ± 6.80	24.20 ± 7.20	Mindfulness-Based Psychological Intervention	Routine Care	24weeks	①
Wang2016 (27)	China	RCT	43	44	25.00 ± 7.00	23.80 ± 6.80	Mindfulness-Based Psychological Intervention	Routine Care	24weeks	①
Tang2021 (28)	China	RCT	29	30	48.13 ± 13.29	47.16 ± 11.99	Mindfulness-Based Cognitive Intervention	Routine Care	8weeks	①
Song2024 (29)	China	RCT	47	47	39.64 ± 4.98	40.19 ± 5.36	Mindfulness-Based Psychological Intervention	Routine Care	12weeks	②
Liu2024 (30)	China	RCT	38	35	26.18 ± 4.91	26.83 ± 4.79	Mindfulness-Based Psychological Intervention	Routine Care	8weeks	②
Chao2024 (31)	China	RCT	102	102	44.59 ± 3.69	44.63 ± 3.70	Mindfulness-Based Psychological Intervention	Routine Care	8weeks	②
Ye2023 (32)	China	RCT	40	40	40.27 ± 6.17	40.21 ± 6.17	Mindfulness-Based Cognitive Intervention	Routine Care	6weeks	②
Yang2019 (33)	China	RCT	36	36	–	–	Mindfulness-Based Cognitive Intervention	Routine Care	8weeks	②

①Insight; ②Stigma.

### 3.2 Meta-analysis results

#### 3.2.1 Insight

**Figure 3:**
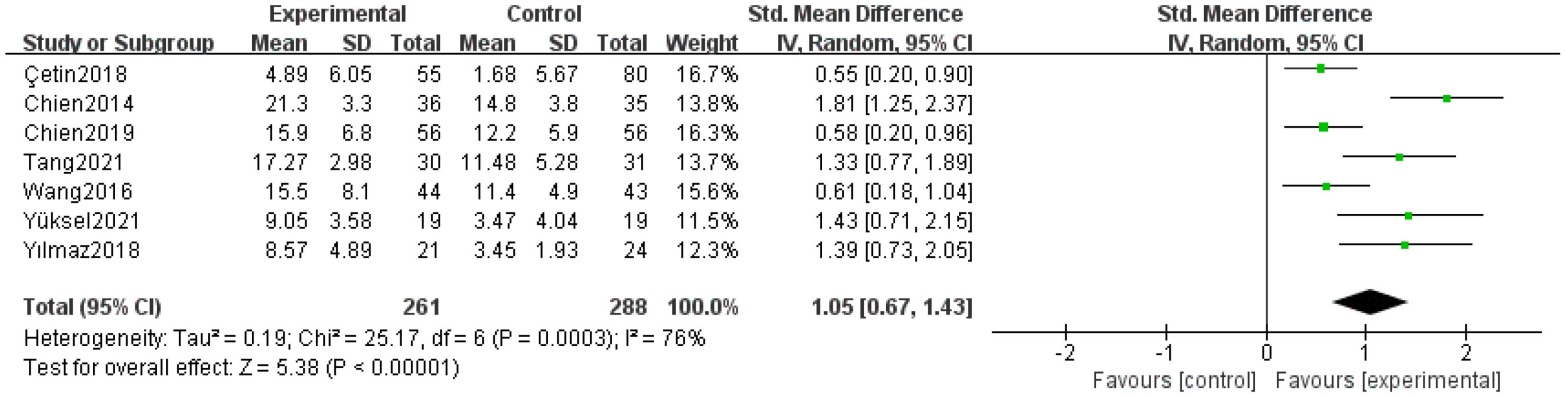
Forest plot of the meta-analysis for insight.

Seven studies (22–28) reported on insight, with significant heterogeneity among the studies (P=0.0003, I²=76%). A random-effects model was adopted after sensitivity analysis. The results indicated that the intervention group demonstrated significantly better insight compared to the control group (SMD=1.05, 95% CI=0.67–1.43, P<0.00001), as shown in **Figure 3**.

#### 3.2.2 Stigma

**Figure 4:**
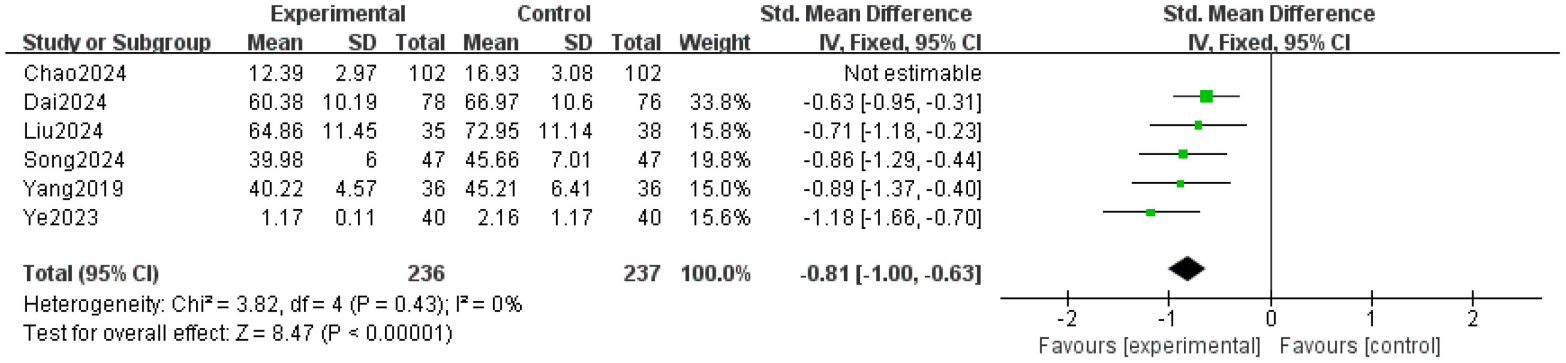
Forest plot of the meta-analysis on stigma.

Six studies (21, 29–33) reported stigma, with significant heterogeneity among the studies (P=0.004, I²=71%). After sensitivity analysis excluding Chao’s study (31), the heterogeneity was reduced (P=0.43, I²=0%), and a fixed-effects model was adopted. The results indicated that the intervention group had significantly lower stigma compared to the control group (SMD=-0.81, 95% CI=-1.00 to -0.63, P<0.00001), as shown in **Figure 4**.

## 4 Discussion

The results of this meta-analysis indicate that mindfulness-based interventions (MBIs) can significantly improve insight in patients with schizophrenia. This finding is consistent with recent studies (34), suggesting that mindfulness training may enhance patients’ illness awareness through multiple mechanisms. Mindfulness emphasizes non-judgmental awareness of present-moment experiences, helping patients more objectively recognize their symptoms and reducing denial or distorted perceptions of the illness. In patients with schizophrenia, impaired illness awareness presents a significant challenge in clinical interventions (35). Mindfulness training, particularly through breathing exercises and body scanning, helps patients differentiate between hallucinations and reality-based experiences, thereby enhancing symptom awareness (36). This enhancement not only deepens patients’ understanding of their illness but also mitigates symptom interference, creating a more favorable foundation for the implementation of other therapeutic approaches. Furthermore, mindfulness enhances metacognitive abilities, enabling patients to reflect on their own thought patterns and reduce “cognitive rigidity,” which is a key factor in impaired insight among individuals with schizophrenia (37). Cognitive rigidity often traps patients in maladaptive thinking patterns, and mindfulness interventions can help them break free from these cycles, thereby improving their responses to symptoms (38). This characteristic of mindfulness training may represent one of its unique advantages in schizophrenia rehabilitation. Compared to traditional cognitive behavioral therapy, mindfulness places greater emphasis on “acceptance” rather than “change,” offering patients a more flexible and non-confrontational approach to symptom management (39). Additionally, the social support provided by mindfulness-based interventions may further strengthen patients’ understanding of their condition, while peer sharing can reduce feelings of isolation and promote the development of illness awareness. Schizophrenia patients often experience heightened stigma due to social exclusion, and the social support network fostered through mindfulness can serve as a significant facilitator in their recovery process (40). Notably, although significant heterogeneity was observed across studies, the overall effect size consistently supports the clinical value of MBIs. These findings suggest that future research should focus on optimizing intervention protocols. In clinical practice, it is recommended to integrate mindfulness training into rehabilitation programs for schizophrenia and adjust intervention intensity according to different disease phases (e.g., acute vs. stable stages).

In terms of stigma, mindfulness-based interventions have demonstrated significant improvement effects. These findings support the notion that mindfulness training can alleviate stigma by reducing self-stigmatization and enhancing psychological resilience (41). Specifically, the “acceptance” principle of mindfulness helps patients reduce the internalization of negative labels. For example, through practices such as the “thoughts as clouds” metaphor, patients learn to observe stigmatizing beliefs from a detached perspective rather than engaging in confrontation. This shift in mindset not only helps improve patients’ emotional states but also equips them with effective tools to cope with social stigma. Studies have further found that mindfulness interventions can reduce emotion-related distress associated with self-stigma (such as shame and anger), thereby decreasing social avoidance behaviors (42). Social avoidance is a common emotional response among patients with schizophrenia, and the application of mindfulness training has effectively alleviated this behavior, consequently improving patients’ social functioning (43). Furthermore, mindfulness interventions combined with psychoeducation can provide scientific knowledge about the illness, correcting misconceptions about schizophrenia and further mitigating the impact of public stigma. Notably, the reduction in stigma may indirectly improve treatment adherence and social functioning, creating a positive feedback loop (44). Treatment adherence is a challenge in the rehabilitation of schizophrenia. Alleviating stigma helps enhance patients’ motivation to engage in treatment, thereby further improving their recovery outcomes. Therefore, reducing stigma is not only crucial for the patient’s emotional state and self-esteem but may also have a profound impact on the overall treatment efficacy. Future research should explore the trajectory of stigma changes in long-term follow-ups and examine the moderating effects of cultural factors (e.g., manifestations of stigma in collectivist societies) on intervention outcomes.

This meta-analysis has several limitations. First, the limited number of included studies and the high heterogeneity—potentially stemming from variations in the specific implementation of interventions and baseline patient characteristics (such as age, gender, and assessment tools)—may affect the generalizability and precision of the results, although sensitivity analysis and the use of a random-effects model partially mitigated this issue. Second, the included literature comprises both RCTs and quasi-RCTs, with the latter carrying a higher methodological risk (e.g., incomplete randomization in sample allocation), thereby reducing the certainty of the findings. Although rigorous quality control measures and sensitivity analyses were employed to evaluate their impact on the conclusions, variations in study quality may still introduce bias. Therefore, future research should rely more on high-quality randomized controlled trials and adopt standardized outcome measures to enhance the reliability and comparability of results. In particular, using uniform measurement tools for assessing stigma and insight would help minimize discrepancies across studies. Moreover, as most included studies were short-term trials, the long-term effects of mindfulness-based interventions remain unclear. While such interventions may demonstrate short-term efficacy, their sustained benefits are not yet fully established. Thus, long-term follow-up studies are essential, and future research should include more high-quality, long-term investigations to verify the enduring effects of mindfulness-based interventions. Lastly, the studies included in this analysis were primarily from China and Turkey, introducing regional limitations. Differences in national, cultural, and social contexts may influence the effectiveness of mindfulness interventions. For instance, manifestations of stigma in collectivist cultures may differ from those in individualistic cultures, potentially affecting intervention outcomes. Future studies should consider cross-cultural and cross-regional comparisons to determine the generalizability and adaptability of mindfulness interventions across diverse cultural and social settings.

This study did not specifically account for potential impacts of publication bias or small-study effects on the results. Although the limited number of included studies precluded formal bias testing, these factors may still have influenced the estimation of effect sizes. Future meta-analyses should incorporate a larger number of studies and include publication bias tests in statistical analyses to further validate our findings.

In summary, compared with the control group, mindfulness-based interventions significantly improved insight and reduced stigma levels in patients with schizophrenia. However, due to the high heterogeneity in some outcome measures, the limited number of included studies, and potential biases, these conclusions should be interpreted with caution. Further large-sample, high-quality, and standardized studies are still needed to validate the long-term efficacy and clinical benefits of mindfulness-based interventions to optimize psychosocial rehabilitation in patients with schizophrenia.

## Data Availability

The original contributions presented in the study are included in the article/supplementary material. Further inquiries can be directed to the corresponding author.
